# Integrating Literature-Based Knowledge Database and Expression Data to Explore Molecular Pathways Connecting PPARG and Myocardial Infarction

**DOI:** 10.1155/2020/1892375

**Published:** 2020-06-01

**Authors:** Rongyuan Cao, Yan Dong, Kamil Can Kural

**Affiliations:** ^1^The Second People's Hospital, Lianyungang, Jiangsu Province 222000, China; ^2^School of Systems Biology, George Mason University, Manassas, VA 20110, USA

## Abstract

Peroxisome proliferator-activated receptor *γ* (PPARG) might play a protective role in the development of myocardial infarction (MI) with limited mechanisms identified. Genes associated with both PPARG and MI were extracted from Elsevier Pathway Studio to construct the initial network. The gene expression activity within the network was estimated through a mega-analysis with eight independent expression datasets derived from Gene Expression Omnibus (GEO) to build PPARG and MI connecting pathways. After that, gene set enrichment analysis (GSEA) was conducted to explore the functional profile of the genes involved in the PPARG-driven network. PPARG demonstrated a significantly low expression in MI patients (LFC = −0.52; *p* < 1.84*e* − 9). Consequently, PPARG could indicatively be promoting three MI inhibitors (e.g., SOD1, CAV1, and POU5F1) and three MI-downregulated markers (e.g., ALB, ACADM, and ADIPOR2), which were deactivated in MI cases (*p* < 0.05), and inhibit two MI-upregulated markers (RELA and MYD88), which showed increased expression levels in MI cases (*p* = 0.0077 and 0.047, respectively). These eight genes were mainly enriched in nutrient- and cell metabolic-related pathways and functionally linked by GSEA and PPCN. Our results suggest that PPARG could protect the heart against both the development and progress of MI through the regulation of nutrient- and metabolic-related pathways.

## 1. Introduction

Myocardial infarction (MI) and afterward heart failure are the significant causes of death and disability in the developed countries, which is characterized by acute myocardial ischemia derived from coronary artery occlusion, myocardial injury, and even necrosis [1–3]. Atherosclerotic plaque rupture with thrombus formation is determined to be the most dominant cause of myocardial infarction, which will result in an acute reduction of blood supply and imbalance in oxygen supply and demand. The prolonged ischemia will cause irreversible myocardial necrosis and heart failure [4–6]. To negate the life-threatening condition, rapid diagnoses and the proper therapy to restore the perfusion are urgent to salvage the jeopardized myocardium.

The members of the peroxisome proliferator-activated receptor (PPAR) family involve PPAR*α*, PPAR*β*/*δ*, and PPAR*γ* (PPARG), which might play vital roles in glucose and lipid metabolism. Among these members, PPARG is enriched in the adipose tissue and widely expressed in extra-adipose tissues, such as the heart, the vascular wall, and the skeletal muscle. PPARG can control the balance between glucose utilization and fatty acid oxidation, which is essential in the energy homeostasis in human myocardia physiology demand and postischemic remodeling [7–9].

As the nuclear hormone receptor superfamily of ligand-activated transcription factors, PPARG could recruit transcription coactivators that are necessary for the initiation of target gene transcription and may also inhibit the development and progress of myocardial infarction [10, 11]. Although some simulators of PPARG have been testified to show a protective effect on the development of myocardial infarction, a systemic literature text mining investigation has been performed to screen the genes and relevant molecular pathways connecting PPARG to myocardial infarction. In this study, literature-based Elsevier Pathway Studio information and expression data retrieved from Gene Expression Omnibus (GEO) were integrated to explore the specific molecules and pathways connecting PPARG and myocardial infarction.

## 2. Materials and Methods

To explore meaningful genetic networks through which PPARG could influence the development and progress of MI, we set up the following rules for the identification of the networks: (1) For each edge (relationship) within a network, there were one or more scientific studies supporting the relationship. (2) A node (gene) demonstrated significant expression changes in the patients of MI.

### 2.1. Identifying PPARG-MI Connection Network

We conducted large-scale literature data mining to identify common genes that were downstream targets of PPARG and also linked to MI. The data was extracted from Elsevier Pathway Studio (http://www.pathwaystudio.com; version 12.3), the database of which is a network of interactions between molecules, processes, and diseases. Each relationship/edge is build based on the fact extracted from the literature by natural language processing (NLP) technology. A manual quality control process was enforced to remove unreliable relationships and relationships with nonspecific polarities by reading the sentences where a relationship was identified. Here, unreliable relationships refer to these with unmatched sentences, which were false positives by the NLP technique. After that, all the entities within the remaining network were tested using a mega-analysis with eight independent MI RNA expression datasets. The process is described as follows.

### 2.2. Selection of Gene Expression Datasets for Mega-analysis

The MI expression datasets were identified within the GEO database (https://www.ncbi.nlm.nih.gov/geo/) [12].The search was conducted using the keyword ‘myocardial infarction,' where 678 studies with series data were identified and downloaded. We made an outline of the metadata of the identified datasets and selected a subset for the mega-analysis with the following criteria applied: (1) The dataset was array expression data. (2) The organism of the dataset was Homo sapiens. (3) The study design was MI case vs. healthy control. (4) The original data and the corresponding format file were downloadable. (5) The sample size was bigger than 10. Eight datasets satisfied the above criteria and were included for the mega-analysis, as shown in Table 1.

### 2.3. Mega-analysis Models

For each gene, the mega-analysis estimated the effect size in terms of gene expression log2 fold-change (LFC). Results from using both the random effects model and fixed effects model were compared [13]. To determine the heterogeneity of the datasets, between- and within-study variance was calculated and compared. When the total variance *Q* was no bigger than the expected value of the between-study variances (df), the model sets the ISq (percentage of the within-study over between-study variance) to zero. In this case, the fixed effects model, instead of the random effects model, will be selected for the mega-analysis. All analyses were performed using MATLAB (R2017a version).

### 2.4. Analysis of Influential Factors

To estimate the possible influence of several factors (e.g., study date, country of origin, and sample size) on the gene expression in MI patients, we conducted a multiple linear regression (MLR) analysis and reported the *p* values for each of these factors.

### 2.5. GSEA and Protein-Protein Connection

To test the functional profile of the genes involved in the PPARG-MI regulation, we conducted a Gene Set Enrichment Analysis (GSEA) [14] against the Pathway Studio pathways and Gene Ontology (GO; http://geneontology.org) terms [15]. The purpose of GSEA was to identify GO terms and Pathway Studio collected pathways enriched with the genes identified within the PPARG-MI network. Additionally, we explored the connections between the genes involved in the PPARG-MI regulation network by using Pathway Studio and constructed the protein-protein connection network (PPCN). Each relationship (edge) within the network was supported by one or more references, which were presented in the Supplementary Material (available here): Ref4PPCN. The PPCN was used to explore the potential functional linkage among the proteins identified within the PPARG-MI network.

## 3. Results

### 3.1. PPARG-MI Regulating Pathway and Mega-analysis Results

**Figure 1:**
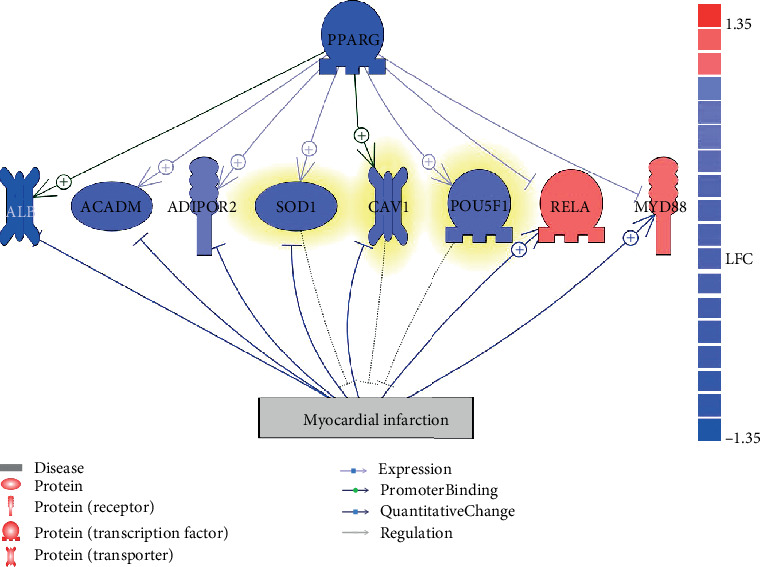
Functional network connecting PPARG and myocardial infarction. Entities in blue are genes with decreased expression levels from the mega-analysis using 8 MI datasets. Entities in red have an increased expression. Entities highlighted in yellow are genes regulating myocardial infarction, and the rest of the genes were targets regulated by myocardial infarction. + represents positive regulation; -| is negative.

Pathway Studio literature text mining identified 30 genes that were promoted by PPARG and also upstream MI regulators (see Supplementary Material: 30 Genes). To identify these genes, we first explored all genes promoted by PPARG; then, we mined all the genes that inhibit MI; after that, we took the overlap and identified these 30 genes. Mega-analysis identified three out of these 30 genes demonstrating a significant decrease in expression levels, including SOD1, CAV1, and POU5F1 (Table 2). These genes were appended in the network connecting PPARG-MI, as shown in Figure 1 (highlighted in yellow).

Following the similar literature text mining approach, we identified 125 genes that were contradirectionally influenced by PPARG and MI (see Supplementary Material: 125 Genes). Out of these 125 genes, three demonstrated significantly increased expression levels in MI patients, including ALB, ACADM, and ADIPOR2 (Table 2). These genes were inhibited in MI while stipulated by PPARG. On the contrary, two genes (e.g., RELA and MYD88) were upregulated in MI patients, which could be suppressed by PPARG (Table 2). These pathways may partially explain the protective role of PPARG in the contradevelopment of MI. Please note that one or more previous studies supported each of these relationships presented in Figure 1. For the details of the supporting references, including relation type, polarity, reference PMID, title, and the sentences where the relation has been described, please refer to Supplementary Material: PPARG_MI_Network.

Mega-analysis showed that the expression levels of PPARG were significantly downregulated in MI patients (LFC = −0.52; *p* value = 1.84*e*-9), which was calculated by using a fixed effects model. This was due to the fact that there was no significant between-study variance (PValue_Q = 0.31) according to the heterogeneity analysis.

Moreover, MLR analysis showed that two factors (country and study age) could significantly influence the expression of PPARG among different studies. For a more detailed description of the mega-analysis results of the nine genes involved in the network presented in Figure 1, please refer to Supplementary Material: Mega-analysis.

### 3.2. GSEA Results and PPI Network

To investigate the biological functions of the nine genes (including PPARG) within the PPARG-MI functional network (Figure 1), a GSEA was executed by using Pathway Studio. A total of eight significantly enriched GO terms (*p* value < 0.005, *q* = 0.005 for FDR) were identified and presented in Table 3, with details made available in Supplementary Material: GSEA. Notably, a majority of the shared GO terms highlighted by the GSEA approach were related to cell metabolic process, nutrient levels, and response to the metal ion, which were implicated with MI [16–18].

**Figure 2:**
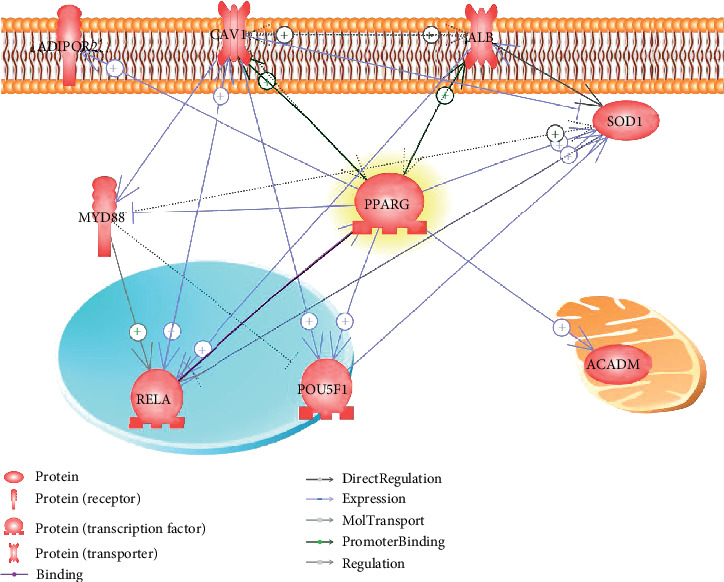
Protein-protein connection networks among the eight genes involved in PPARG-MI signaling pathways. The network was built using Pathway Studio (http://www.pathwaystudio.com). One or more references supported each relationship within the network. + represents positive regulation; -| is negative.

A literature-based PPI network has been constructed and presented in Figure 2. The relation between a pair of genes was identified through literature data mining. For each relationship/edge within Figure 2, there was at least one supporting reference. For the details of these references, please refer to Supplementary Material: Ref4PPCN. The PPCN showed that there were direct physical or indirect functional connection among PPARG and eight of its driven genes.

## 4. Discussion

This study confirmed the downregulation of PPARG in the case of myocardial infarction and revealed multiple pathways through which PPARG could regulate the development of myocardial infarction. Our results shed light on the understanding of the PPARG-MI association, suggesting PPARG as a potential therapeutic target for the treatment of myocardial infarction.

Among the eight genes identified to be driven by PPARG, ALB could be utilized as a monitor biomarker, as a low level of serum ALB is associated with increased risk of coronary artery disease and myocardial infarction [19]. ACADM could be a rate-limiting factor for the initial step of the mitochondrial fatty acid beta-oxidation catalyzation, which plays a vital role in myocardial infarction and diabetic cardiomyopathy [20].

The other six genes may be involved in the functional recovery and cellular protection involved in myocardial infarction. SOD1 overexpression, RELA blockade, and diminished MyD88-mediated inflammation can enhance functional and metabolic recovery and greatly decreased myocardial infarction [21–23], while ADIPOR2 is required for revascularization [24]. On the other hand, HIF-2*α* and POU5F1 (OCT4) could collaboratively promote the survival and differentiation of embryonic-like mesenchymal stem cells in myocardial infarction to repair the damaged myocardia [25]. It is worth to note that downregulated pulmonary CAV-1 expression subjected to myocardial infarction may lead to STAT3/Cyclin pathway activation, pulmonary hypertension, and lung structural remodeling development [26]. All of this evidence indicates that PPARG not only works in the progression of myocardial infarction but also plays a role in the functional recovery and cellular protection of myocardial infarction.

In addition to the exogenous activators, PPARs can also be activated by endogenous secreted ligands, such as free fatty acids or prostaglandins. It is not surprising to find that a majority of the shared pathways highlighted by the GSEA approach are related to cell metabolic process and nutrient levels, which are also implicated in the development of myocardial infarction. It is worth noting that, although detailed information should be deciphered, mitochondrial fatty acid beta-oxidation catalyzation rate limited by ACADM might be the vital energy pathway mediated by PPARG.

The PPCN showed that, besides the relation between PPARG and its eight driven genes (Figure 1), the majority of the eight genes (6 out of 8) were physically or functionally linked to each other (Figure 2). Especially, five out of the rest seven genes were connected to SOD1; the overexpression of which enhances functional and metabolic recovery and significantly decreases MI [21]. These functional connections (Figure 2) suggested that the genes connecting PPARG and MI may be also functionally linked to each other.

In this study, we propose an integrated analysis employing both literature-based knowledge database and expression data to explore the functional connection between PPARG and MI. This approach could help the exploration of the crucial genes and pathways further to decipher the association of factors in interest with a particular disease. Both the metabolic-and nutrient-associated pathways involved in the development and progress of myocardial infarction can be regulated by PPARG, which indicates that PPARG might be utilized as an essential target in myocardial infarction treatment.

This study has several limitations that need to be addressed in further work. First, the PPARG-MI connecting network was constructed using Pathway Studio only. More data sources should be employed to explore more potential relationships. Second, we used array data to study the expression variation of PPARG and its driven genes. Expression by RNA sequencing may provide higher resolution in studying the expression profile.

## 5. Conclusion

Literature-based knowledge database and expression data integration may significantly promote the illustration of the relevant mechanism involved in PPARG-mediated myocardial infarction protection.

## Figures and Tables

**Table 1:** The eight MI expression datasets selected for mega-analysis.

Dataset GEO ID	No. of controls	No. of cases	Country	Study age	Sample organism
GSE24519	4	34	Italy	3	Homo sapiens
GSE24591	4	34	Italy	3	Homo sapiens
GSE34198	48	49	Czech Republic	6	Homo sapiens
GSE48060	21	31	USA	6	Homo sapiens
GSE60993	7	10	South Korea	5	Homo sapiens
GSE60993	7	17	South Korea	5	Homo sapiens
GSE62646	14	84	Poland	6	Homo sapiens
GSE66360	50	49	USA	5	Homo sapiens

Note: Study age = current year − year of study + 1.

**Table 2:** Mega-analysis results of the eight genes involved in the PPARG-MI regulatory network.

Gene name	Random-effects model	No. of studies	LFC	*p* value	No. of samples	Country	Study age
SOD1	0	4	-0.28	0.048	0	0	1.00
RELA	0	4	0.28	0.008	0	0	1.00
POU5F1	1	5	-0.23	0.041	1.00	2.00*E*-09	2.00*E*-13
MYD88	0	3	0.23	0.047	0	0	1.00
CAV1	0	7	-0.24	0.0050	0.12	0.013	0.070
ALB	1	6	-1.35	0.0050	0.40	0.022	0.0030
ADIPOR2	0	6	-0.16	0.031	0.22	6.00*E*-04	1.00
ACADM	0	6	-0.32	0.0034	0.0056	0.000329	0.99328
PPARG	0	5	-0.52	2*e*-09	1	6.75*E*-11	5.28*E*-10

**Table 3:** The GO terms enriched with nine genes within the PPARG-MI functional network.

Name	No. of entities	GO ID	Overlap	*p* value	Jaccard similarity
GO: response to nutrient	370	0007584	7	2.94*E*-07	0.019
GO: response to nutrient levels	730	0031667	7	1.53*E*-05	0.0096
GO: response to extracellular stimulus	761	0009991	7	1.53*E*-05	0.0092
GO: response to inorganic substance	803	0010035	6	0.0011	0.0074
GO: regulation of small molecule metabolic process	422	0062012	5	0.0016	0.012
GO: regulation of lipid metabolic process	441	0019216	5	0.0016	0.011
GO: cellular response to external stimulus	451	0071496	5	0.0016	0.011
GO: response to metal ion	533	0010038	5	0.0033	0.0093

## Data Availability

The data in our study are available from the corresponding author upon reasonable request.

## Supplementary Materials

Supplementary Materials Supplementary materials were provided for this study (an excel file), including the following worksheets: PPARG_MI_Pathway, 30 Genes, 125 Genes, Mega-analysis, GSEA, and Ref4PPCN.
